# Transport of Veterinary Antibiotics in Farmland Soil: Effects of Dissolved Organic Matter

**DOI:** 10.3390/ijerph19031702

**Published:** 2022-02-02

**Authors:** Lanre Anthony Gbadegesin, Xiangyu Tang, Chen Liu, Jianhua Cheng

**Affiliations:** 1 Key Laboratory of Mountain Surface Processes and Ecological Regulation, Institute of Mountain Hazards and Environment, Chinese Academy of Sciences, Chengdu 610041, China; lanrex2010@gmail.com; 2 International College, University of Chinese Academy of Sciences, Beijing 100049, China; 3 State Key Laboratory of Subtropical Silviculture, Zhejiang A&F University, Hangzhou 311300, China; chengjh@zafu.edu.cn

**Keywords:** fate, interactions, manure, spectroscopic analyses, transport model

## Abstract

The application of manure as a fertiliser to farmland is regarded as a major source of veterinary antibiotic (VA) contamination in the environment. The frequent detection of such emerging contaminants and their potential adverse impacts on the ecosystem and human health have provoked increasing concern for VA transport and fate. Extrinsic dissolved organic matter (DOM) may be introduced into farmland soil along with Vas, and thus exert significant effects on the transport of VAs via hydrological processes upon rainfall. The leaching of VAs can be either enhanced or reduced by DOM, depending on the nature, mobility, and interactions of VAs with DOM of different origins. From the aspect of the diversity and reactivity of DOM, the state-of-the-art knowledge of DOM−VA interactions and their resulting effects on the sorption−desorption and leaching of VAs in farmland soil was reviewed. Spectroscopic techniques for examining the extent of binding and reactive components of DOM with VAs are summarized and their usefulness is highlighted. Models for simulating VA transport under the effects of DOM were also reviewed. It is suggested that distinct impacts of DOM of various organic fertiliser/amendment origins should be considered for predicting the transport of VAs in farmland soil.

## 1. Introduction

**Figure 1:**
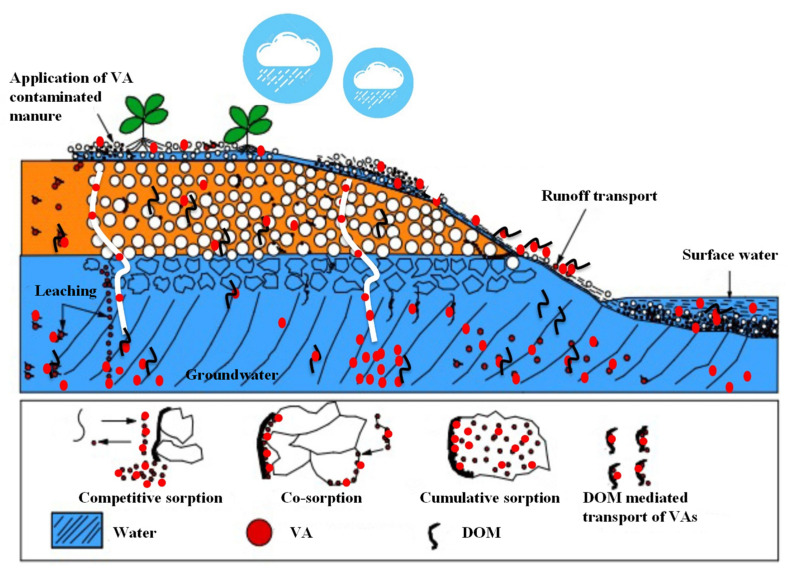
Transport paths of veterinary antibiotics (VAs) via hydrological processes and the mechanisms of potential interactions of VAs with dissolved organic matter (DOM).

Antibiotics are popularly used in animal husbandry as therapeutics, and to improve livestock productivity. The global average annual antibiotic consumption in cattle, chicken, and pigs was 45 mg kg^−1^, 148 mg kg^−1^, and 172 mg kg^−1^, respectively, and the global total antibiotic consumption in food animals was projected to increase by 67% by 2030 [1]. A significant quantity (40–90%) of active and unchanged forms of veterinary antibiotics (VAs) administrated to animals are excreted with the urine and faeces of the animals [2,3]. An investigation on the environmental fate of 36 frequently detected antibiotics, including sulfonamides (SAs), tetracyclines (TCs), fluoroquinolones (FQs), macrolides (MLs), β-lactams, and others, was conducted in China in 2013. The result showed that 84% of the total antibiotic excretion (54,000 tons) was excreted by animals, mainly pig (44.4%), chicken (18.8%), and other (20.9%), and 54% of the total antibiotic emission (53,800 tons) was received by soil compartments [4]. The application of animal manure as a fertiliser in farmland is a major pathway for VA entrance into the environment (Figure 1) [5,6]. It should be noted that the presence of VAs in manure applied to farmland could instigate the elevated occurrence of antibiotic-resistant genes (ARGs) in soil, which are harboured by pathogenic and/or non-pathogenic microbes [7,8,9,10,11,12]. Moreover, residual antibiotics in farmland soil and hydrologically connected water bodies could impair ecosystem functions and cause severe contamination of food and drinking water supplies [13]. As such, a full understanding of VA transport and fate in soil is of high relevancy with respect to their environmental risk in manured farmland.

Dominant paths of VA transport in farmland soil are runoff and leaching, both of which are dependent on VA sorption to soil, soil hydraulic properties, and precipitation characteristics [14,15,16,17]. It is worth noting that the sorption of VAs to the soil matrix can not only reduce their mobility and availability, but also protect antibiotics from degradation [18]. Preferential flow through soil macropores may lead to rapid transport of VAs and other organic pollutants in the subsurface environment [19,20]. In addition, natural occurring colloids (e.g., clays, metal oxides, and particulate organics) in farmland soil can act as carriers of Vas, and thus may lead to enhanced VA transport through preferential pathways (e.g., macropores, cracks, and fractures) [21,22,23]. Notably, the transport of VAs in soil can be strongly influenced by dissolved organic matter (DOM) [24,25,26,27,28,29,30,31,32,33,34], which is operationally defined as the fraction of organic matter that can pass through a 0.45 µm filter and is present in the aqueous phase [35,36,37]. It is regarded as the most active component of soil organic matter due to its diverse chemical structures, functional groups, and molecular sizes [36,38,39]. DOM is ubiquitous in the terrestrial environment and can be transported from farmland soils to water bodies through leaching and runoff processes [40,41]. In farmland soils, in addition to soil organic matter, decaying crop straws are the primary natural source of DOM [42]. Furthermore, various soil amendments (e.g., animal manure, biosolid, and biochar) applied to improve soil fertility can also release a substantial amount of DOM into the soil [43,44]. For instance, the application of animal manure may lead to the enrichment of both VAs (excreted with manure) and DOM in the soil [17,45,46], and thus strong interactions between VAs and DOM, which could play a significant role in altering the transport and fate of VAs in the soil [26,47,48]. To date, the state-of-the-art knowledge of DOM−VA interactions and their impacts on VA transport have not yet been systematically reviewed.

In this article, a literature review was conducted using different databases (Google Scholar, ScienceDirect, and Web of Science), as well as relevant keywords regarding the following aspects: (1) sources and occurrences of DOM in soil and its influence on sorption-desorption and transport of VAs; (2) main mechanisms of DOM-VA interactions; and (3) current mathematical and conceptual models used to describe VAs transport in the presence of DOM. Lastly, future research directions were recommended.

## 2. Sources and Occurrences of DOM in Soil

In farmland, DOM leached from topsoil can be sorbed and thus stored in deep subsoil with a relatively low organic matter content, which is recognized as a carbon reservoir [49,50,51,52]. DOM of autochthonous and allochthonous origins differ in properties and biogeochemical behaviour [53,54]. Joint use of multiple techniques to measure DOM properties is necessary, as no single technique is capable of fully characterizing DOMs [55]. Notably, florescence spectroscopy has gained wide acceptance as a technique for measuring DOM moieties in farmland soil (Table S1). Humic-like and tyrosine-like molecules were found to be the major components of DOM in all the dissimilar farmland soils collected from four climate regions in China [56].

Organic soil amendments, such as crop straw, animal manure, compost, biochar, and sewage sludge, can supply the soil with DOM of diverse compositions and contents (e.g., [54,57,58]), as shown in Table S1. For instance, crop straw can release a large amount of DOM into farmland soil [59]. According to spectroscopy investigations, fulvic-like, tyrosine-like, humic-like, and tryptophan-like fluorophores are vital components of straw derived DOM (Table S1) [57,60,61]. Characteristics of straw derived DOM, including aromaticity, hydrophobicity, and molecular weight, could vary with straw humification time and crop type [59]. Specifically, hydroxyl, carboxyl, amine, and aliphatic C–H are the functional groups of straw-derived DOM that can interact with the soil and organic pollutants [59,62]. Animal manure DOM show diverse physicochemical characteristics and chemical structures, depending on the feedstock and composting method [33,63,64]. DOM of different animal manure origins may differ in interactions with VAs and thus differ in their effects on their transport in soil [31,65].

DOM interacts with the reactive surface of soil particles, and its transport and leaching behaviour may vary with soil type, landscape, slope, and varying climatic factors [35,66,67]. A high affinity of DOM for soil clay surface constrains its movement in the soil [68]. A positive relationship of DOC sorption with iron oxides and the specific surface area of clay minerals (kaolinite < illite < smectite) was reported [69]. The presence of polyvalent exchangeable cations could reduce DOM leaching by enhancing its sorption, mainly through cation bridging between negatively charged clay surfaces and negatively charged anionic functional groups of DOM [51,70,71,72]. It has been recognized that DOM mobility may affect the fate and transport of antibiotics in soil [25,33,73,74]. 

## 3. Effect of DOM on the Sorption-Desorption of VAs

DOM is one of the major factors that influence the sorption and transport of VAs in soil [25,28,31,33,34]. Table 1 summarises the effects of DOM on the sorption−desorption of VAs reported in the literature. Various mechanisms, including covalent bonding, π−π interaction, complexation reaction, electrostatic interaction, van der Waals force, hydrophobic partitioning, and pore entrapment, may co-occur in the sorption−desorption process of VAs in a soil−DOM system [24,26,30,31]. The interactions between DOM and VAs strongly depend on the solution pH and ionization state of VAs [7,30]. The sorption−desorption process may become more complex in the presence of DOM, as it could provide abundant functional groups that are reactive with soil surfaces and VAs.

DOM may have opposite effects on VA sorption to soil [73] (Figure 1). For instance, the addition of manure DOM was reported to decrease sorption *K*_f_ (the sorption coefficient of the Freundlich model) of chlortetracycline, tylosin, and sulfadiazine to soil, while it increased the sorption *K*_f_ of sulfamethazine [75,95]. DOM can form complexes with VAs and the complexes may be sorbed onto soil surface, which is a process termed “co-sorption”; DOM may also occupy the active sites on the soil matrix and thus provide additional sorption sites for VA sorption through its multiple moieties, which is a process termed “cumulative sorption” [62,96,97]. For instance, significant increases in the sorption *K*_d_ (the distribution coefficient of the linear model) of ciprofloxacin and tetracycline caused by coating the soils with humic acids (HAs) were observed [98]. A marked decrease in the sorption *q*_max_ (the maximum sorption capacity of the Langmuir model) of tetracycline from 5241 mg kg^−1^ to 1274 mg kg^−1^ was observed after DOM had been extracted from the soil [81]. It was found that the addition of straw derived DOM could cause an increase in the sorption *q*_max_ of sulfamethoxazole by 2.6 times [62]. Contrastingly, DOM may restrict VAs from being sorbed to the soil surface by inducing a masking effect that repels or desorbs VAs from the solid phase into the solution phase, which is a process termed “competitive sorption” [51,99,100]. For instance, the pre-sorption and co-introduction of DOM in soils with low organic matter contents (<0.15%) was found to result in enhanced desorption of sulfapyridine. Similarly, a decrease in the sorption *K*_f_ of florfenicol by about 23% was observed upon the addition of manure-derived DOM, as compared to treatments without DOM [34]. Overall, the varying sorption−desorption behaviours of VAs in the presence of DOM are dependent on the physicochemical properties (e.g., molecular weight, aromaticity, and hydrophobicity) of DOM and soil properties, as well as the solution condition (e.g., pH and salts) [56,62].

## 4. Effect of DOM on the Transport of VAs

**Figure 2:**
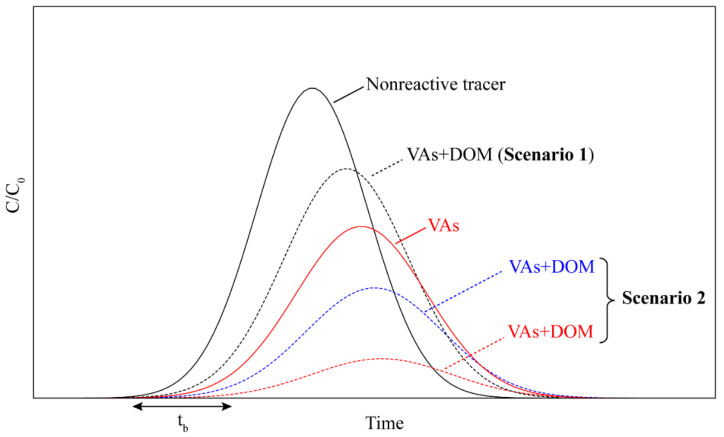
Schematic diagram showing contrasting hypothetical breakthrough curves (BTCs) of nonreactive tracer and veterinary antibiotics (VAs) in the presence and absence of dissolved organic matter (DOM) through homogeneous soil columns. Scenario 1: facilitated transport of VAs through mechanisms such as co-transport, competitive sorption, and colloid facilitated transport. Scenario 2: retardation of VAs through mechanisms such as co-sorption, cumulative sorption, complexation reaction, and straining/pore-entrapment of mobile colloids bearing VAs. *t*_b_ and *C*/*C*_0_ are breakthrough time and relative concentration, respectively.

DOM can be regarded as reactive and mobile organic colloids. The presence of DOM colloids may enhance the transport of VAs in soil via two main mechanisms: DOM can form complexes/association with VAs in a solution and thus increase the desorption of VAs from solid phase into pore water. On the other hand, DOM may also be preferentially sorbed to soil particles and thus occupy the available sorption sites, leading to increased release and leaching of VAs from the soil system into the groundwater [33,34]. For instance, DOM of different manure sources was reported to facilitate sulfamoxole leaching in the presence of highly mobile manure DOM, mainly as a result of the competitive sorption of DOM with sulfamoxole for the sorption sites [33]. Similarly, co-transport of dairy manure DOM and chlortetracycline reportedly increased the mass recovery (from 2.1% to 4.3%) and reduced the retardation factors (from 890 to 371) of chlortetracycline, which was also attributed to competitive sorption [95]. Aside from the DOM of a manure origin, soil-derived HA can also significantly enhance the transport of tetracycline by more than 10% through competitive sorption [101]. Likewise, the recovery rate of ciprofloxacin was reported to increase by 32.7% in soil coated with soil-derived HA, while tetracycline recovery increased from 13.8% to 33.2% in the presence of soil colloids [98]. Contrastingly, retardation of sulfadiazine and sulfamethoxypyridazine was observed when manure DOM was added, due to their high sorption affinity to the sorbed DOM on soil [33]. In addition, the pre-loading of natural DOM in soil columns could facilitate the transport of nalidixic acid (a quinolone antibiotic), which was attributed to enhanced retardation through hydrophobic and π−π interactions [102]. DOM could have opposite effects on the breakthrough curve of VAs in a homogenous repacked soil column, depending on the nature of the interactions of DOM with soils and VAs (Figure 2). 

Among the VA groups, SAs and TCs are ubiquitous in surface water and groundwater due to their hydrophilicity and high mobility, although their interactions with DOM can alter their fate and sorption to the solid surface. For instance, a field investigation in an intensive agricultural and livestock production area showed that remarkable concentrations of SAs in groundwater were associated with aliphatic and unsaturated oxygen-poor constituents of DOM, while the presence of TCs were related to the unsaturated high-oxygen-rich constituents of DOM [103]. Manure DOM with a high content of hydrophobic compounds, protein C, SUVA_280_, and a low C/N ratio was reported to enhance the leaching of sulfamoxole [33]. These studies highlighted the significance of the chemical nature of DOM and VAs in DOM−VA interactions and VA transport in soils. 

In addition to leaching, offsite effects of VAs via runoff transport have attracted considerable environmental concerns. As illustrated in Figure 1, antibiotics released from manure-fertilised farmlands after rainfall or irrigation can be transported via runoff to low-land areas and surface waters. VAs, including TCs, SAs, and MLs, have been detected in runoff and surface water [14,104,105]. For example, in a tramline plot experiment, 703.2 and 71.1 µg L^−1^ of SAs and TCs, respectively, were detected in runoff water from the plots treated with animal slurry [105]. Likewise, oxytetracycline and chlortetracycline were detected in runoff transport from an irrigated pasture in ranges of 1–700 and 1–1300 ng L^−1^, respectively; furthermore, contents of VAs in the range of 1–24 µg kg^−1^ were detected in the upper 5 cm soil below the manure pat [14].

## 5. Characterisation of DOM−VA Interactions in Soil−Water Systems

Spectroscopic analyses of DOM−VA binding interactions have been undertaken in a few previous studies.

### 5.1. Functional Properties of DOM

Hydrophobicity and molecular weight are two important functional properties of DOM affecting its interactions with VAs in the environment [106]. DOM can be operationally fractionated into two major parts based on hydrophobic−hydrophilic characteristics [106,107]. XAD resin is a broadly used technique [107,108] to separate DOM based on their sorption properties. Hydrophobic−hydrophilic fractions of DOM were found to play a significant role in governing the fate of organic pollutants in soil, including pharmaceuticals [106].

Molecular weight distribution of DOM is an essential property in monitoring of pollutant distribution, persistence, and transport in natural water and soil [109,110]. Size exclusion chromatography (SEC) can be used in combination with high-performance liquid chromatography (HPLC) to fractionate DOM based on molecular size [111,112]. DOM contains high molecular weight substances (humic and fulvic acid), as well as low molecular weight substances (proteins, organic acids, carbohydrates, and other compounds) [108]. Diverse molecular properties of DOM are responsible for its significant reactive nature in the environment [111,113]. For example, the binding capacity of large size DOM fractions (>100 kDa and 10–100 kDa) to FQs was found to be lower than that of smaller size DOM fractions [114]. Likewise, the binding capacities of DOM size fractions to TCs (tetracycline, oxytetracycline, and chlortetracycline) in the order of 30 kDa–0.45 µm > 5–30 kDa > below 1 kDa > 1–5 kDa was observed, indicating that the fractions of 30 kDa–0.45 µm and 5–30 kDa could facilitate the transport of TCs [27].

### 5.2. Spectroscopic Properties of DOM

Spectroscopic methods have been extensively used for the chemical and structural characterisation of DOM due to their accuracy and ability to give a detailed molecular structure of DOM [44,115], and have shown much usefulness in elucidating the interactions between DOM and VAs, especially in water [29,31,32,81,89,116,117].

#### 5.2.1. Ultraviolet and Fluorescence Spectroscopy of DOM

Ultraviolet and visible (UV−VIS) absorbance has been widely used to characterise DOM as a non-destructive and inexpensive method [118]. It reflects the carboxylic and aromatic chromophores of DOM based on the absorbance wavelength [113], and absorbance ratios such as A_254_/A_204_, A_254_/A_436_, and A_250_/A_365_ are correlated with DOM reactive properties and thus valuable for DOM characterisation [31,113,119,120]. UV−VIS absorbance is often used together with a three-dimensional fluorescence excitation−emission matrix (3D-EEM) to comprehensively describe DOM−VA interactions [31,89,117]. 

Fluorescence EEM spectroscopy provides valuable insights into DOM−VA interactions by revealing changes in the fluorescence peak intensities of interacting compounds (Table 2). EEM fluorescence peaks may contain five regions, which represent tyrosine, tryptophan, and HA-like organics [121], and were debatably attributed to carboxyl, phenolic hydroxyl, or carbonyl groups, respectively [122]. These fluorescent components of DOM play crucial roles in DOM−VA interaction [123]. Fluorescence quenching may occur when DOM interacts with the VA molecule and suppresses its energy emission [124]. Such a quenching process may lead to energy transfer or to the formation of a ground-state complex, which is commonly referred to as dynamic or static quenching, respectively [82,123]. Static quenching implies that DOM−VA binding or complexation leads to a significant decrease in freely dissolved VAs in soil, while dynamic quenching results from charge transfer that occurs when fluorophore and quencher collided [26,29,31,117]. The complexation and binding properties of DOM−VA interactions were found to be highly related to the proportion of DOM fluorescent components [31,117].

Different DOM components behave differently in their binding interactions with VAs. The protein-like components of DOM can form strong binding interactions with VAs [24,82,127]. For example, compared to humic-like components, the protein-like (tyrosine and tryptophan) component of DOM from various sources binds more strongly with TCs, sulfamethazine, sulfaquinoxaline-sodium, oxytetracycline, and erythromycin, through multiple molecular binding sites accessible in this component [24,81,117]. As such, the fluorescence EEM spectroscopy technique has gained wide acceptance for studying DOM−VA interactions [24,81,114,115,127,128,129,130].

#### 5.2.2. FTIR and NMR Spectroscopy

The DOM functional groups participating in DOM−VA interactions can be investigated using FTIR in combination with other spectroscopy methods [29,31,81,89]. Moreover, FTIR spectroscopy can be used to reveal the non-fluorescent components, including the polysaccharide-like and aliphatic compounds of DOM, that are involved in DOM−VA interactions [29,31,81]. The aromatic ring and double bond region were found to be the primary binding sites in DOM−VA interactions [31]. The FTIR spectra of HA−FQ interaction showed that O-H, C-H, and -COOH were important functional groups involved in the interactions, while N-H involvement was observed only for certain FQs [31]. Similarly, amide I and II, aromatics, and aliphatic were found to be the main functional groups of DOM responsible for the complexation of TCs in DOM−TC interactions [89]. Therefore, FTIR spectra can be used to reveal the extent of DOM−VA binding associated with DOM functional groups. In many studies (as shown in Table 2), various spectroscopic methods were used in combination to provide details of DOM−VA binding interactions at a molecular scale (e.g., [31,81,89]).

Nuclear magnetic resonance (NMR) spectroscopy is a simple, sensitive, and effective technique that can be used to monitor the molecular changes at the DOM−VA interaction sites [31,121]. For example, in the ^1^H NMR spectra, gradually weakened and broadened proton signals of FQs with increasing HA concentration were observed; a chemical shift occurred mainly in the aromatic and double region (≥6.9 ppm), while a diamagnetic shift was seen in the aliphatic regions (1–4.7 ppm), reflecting a strong interaction between HA and FQ [31]. Carboxyl and phenolic hydroxyl groups of HA and -N(CH_3_)_2_ groups of TCs were engaged in HA−tetracycline antibiotic interactions [121]. Therefore, NMR is useful and can provide rich structural information with a higher accuracy and sensitivity about DOM−VA interactions.

#### 5.2.3. High-Resolution Mass Spectrometry (HRMS) of DOM

High-resolution mass spectrometry (HRMS) is a powerful tool that has been widely used to elucidate the DOM structural composition [103,131,132,133]. HRMS can provide a wide dynamic range of screening and reliable quantitative DOM molecular nature at a higher resolution. It can accurately identify, quantify, and determine the molecular composition of DOM [103,131,134]. Among the HRMS instruments, Fourier transform ion cyclotron resonance mass spectrometry (FTICR-MS) is widely used. In recent years, Orbitrap HRMS has become popular as a valid alternative to FTICR-MS. For instance, Orbitrap HRMS was found useful and was employed for identifying the potential DOM components associated with VAs in groundwater [103].

Integration of spectroscopic methods in studying DOM−VA interactions can provide an accurate understanding of the DOM molecular structures, functional groups, and predominant components involved in binding interactions with VAs, which could stimulate the development of novel techniques for the assessment of environmental risk and remediation of VA pollution.

### 5.3. Binding Stability of DOM-VA Interactions

The binding stability of the DOM−VA interaction is a key factor affecting the transport behaviour of VAs in the presence of DOM. The Stern−Volmer equation is popularly used to fit the quenching data for quantitative description of DOM−VA interactions and to estimate parameters including the quenching rate constant [24]. Fluorescence decay curves plotted from the fluorescence intensity data can provide more valuable information on the quenching mechanism [31]. With the site-binding equation, the binding constant (*K*_b_) and number of binding sites (*n*) in DOM−VA interactions can be obtained [31,102,135]. In addition to the Stern−Volmer equation and site-binding equation, the magnitude and signs of thermodynamic functions (e.g., Van’t Hoff equation) can be used to explore the major forces contributing to HA−quinolone antibiotic binding stability [32,116].

It has been recognized that increasing the temperature may lead to a greater extent of collisional quenching, while decreasing the temperature may result in the formation of more non-fluorophore complexes [29,136,137]. In a study on interactions between FQs with DOM, the Stern−Volmer quenching constant and the binding constant was found to increase with the decreasing temperature, which indicates strong static quenching interactions between FQs and HA [31]; negative values of enthalpy (∆*H*^0^) and entropy (∆*S*^0^) were observed, which indicate a well-organised conformation of HA−quinolone antibiotic complexes involving van der Waals forces, hydrogen bonding, and electrostatic interaction; however, positive ∆G^0^ values were obtained, which indicate the binding processes were exothermic and not thermodynamically favourable [31]. In a study on the binding interactions of six FQs with HA over wide temperature ranges, it was found that all the FQs were quenched through a static mechanism, and the equilibrium binding constants were <1 (*K*_b_: 0.0332 to 0.172 at 25 °C), indicating that most of the antibiotics remained unbound in the system; negative values of ∆*H* and ∆*S* were obtained, implying that hydrogen bonding was involved in HA−quinolone antibiotic interactions [116]. In a study on the binding characteristics of HA−quinolone antibiotic using the Ryan and Weber model, the stability constant (*K*_M_) ranged from 0.86 to 4.07 L mg^−1^ and higher *K*_M_ values were associated with a higher total ligand concentration of HA [114].

In particular, dynamic quenching was found to contribute significantly to the overall fluorescence quenching of ofloxacin by HA [29]. The formation of complexes by tyrosine and tryptophan with TCs (protein-like-TCs) was stronger than that of the complexes formed by humic-like substance, as indicated by the effective quenching constant values [89]. The binding stability of the DOM−VA interaction is a significant factor that may moderate the fate of VAs in the presence of DOM.

## 6. Modelling the Transport of VAs under DOM Impact

There are two categories of models that can be used to simulate the transport of antibiotics in soil as affected DOM. These models have been largely applied for homogeneous soils so far.

### 6.1. Transport Models

#### 6.1.1. Chemical Nonequilibrium Transport Models

The chemical nonequilibrium models are built on the assumption that, in homogenous soils (such as repacked soil columns), chemical factors are responsible for the nonequilibrium transport behaviours of reactive solutes [138,139]. The simplest form of the chemical nonequilibrium transport model is an advection−dispersion equation coupled with one kinetic site sorption model. One kinetic site sorption model assumes that the sorption of reactive solute occurs kinetically in a uniform transport domain, and it is usually described using the first-order equation. Thus, the conventional advection−dispersion equation is modified to include a first-order equation that describes the one-site sorption model, as given below [92]:

(1) $$\frac{\partial nc}{\partial t}+\rho \frac{\partial S}{\partial t}=\frac{\partial }{\partial z}\left(\theta D\frac{\partial c}{\partial z}\right)-\frac{\partial qc}{\partial z}$$

(2) $$\rho \frac{\partial S}{\partial t}=\theta k_{\mathrm{att}}\psi c-k_{\det}\rho S$$

(3) $$\psi =1-\frac{S}{S_{\max}}$$

where *n* is the porosity (L^3^ L^−3^); *t* is time (T); *c* is the solution concentration (M L^−3^); *S* is the sorbed concentration on kinetic sorption sites (M M^−1^); *θ* is the water content (L^3^ L^−3^); *D* is the hydrodynamic dispersion coefficient (L^−^^2^ T^−^^1^); *z* is the coordinate parallel to flow (L); *ρ* is the bulk density (M L^−3^); *q* is the flow rate (L T^−1^); *k*_att_ and *k*_det_ are the first-order attachment rate and detachment rate, respectively (L^−1^); *ψ* is a dimensionless function that describes the Langmuirian blocking; and *S*_max_ is the saturated sorption capacity (M M^−1^).

The one kinetic site sorption model can further be extended by conceptually dividing the sorption sites into two fractions. It is assumed that instantaneous sorption occurs on one fraction of the sites (Type 1 sites), while sorption on the other fraction (Type 2 sites) is kinetically controlled:

(4) $$S=S^{e}+S^{k}$$

where *S*^e^ and *S*^k^ (M M^−1^) are fractions of the sorption sites assumed to be instantaneous (Type 1) and first-order kinetic rate (Type 2), respectively.

Accordingly, the conventional advection−dispersion equation is modified to be the two sorption site model as follows:

(5) $$\frac{\partial c}{\partial t}+\rho \frac{\partial S^{e}}{\partial t}+\rho \frac{\partial S^{k}}{\partial t}=\frac{\partial }{\partial z}\left(\theta D\frac{\partial c}{\partial z}\right)-\frac{\partial qc}{\partial z}-\phi$$

(6) $$S^{e}=f_{e}K_{d}c$$

(7) $$\rho \frac{\partial S^{k}}{\partial t}=\alpha_{k}\rho \left(S_{e}^{k}-S^{k}\right)-\phi_{k}$$

(8) $$S_{e}^{k}=\left(1-f_{e}\right)K_{d}c$$

where *f*_e_ is the fraction of sorption sites in equilibrium with liquid-phase (dimensionless), *K*_d_ is the linear sorption coefficient (L^3^ M^−1^), *α*_k_ is the first-order rate coefficient associated with the kinetic site (T^−1^), and *φ* is the sink term (n L^−3^ T^−1^).

The two sorption site model (Equation (5)) can be transformed into the two kinetic site model by the concept that sorption could occur kinetically on both fractions of the sorption sites and proceed at different rates (Equation (9)), which can be written as:

(9) $$\frac{\partial c}{\partial t}+\rho \frac{\partial S_{1}^{k}}{\partial t}+\rho \frac{\partial S_{2}^{k}}{\partial t}=\frac{\partial }{\partial z}\left(\theta D\frac{\partial c}{\partial z}\right)-\frac{\partial qc}{\partial z}-\phi$$

(10) $$\rho \frac{\partial S_{1}^{k}}{\partial t}=k_{a1}\theta c-k_{d1}\rho S_{1}^{k}-\phi_{k1}$$

(11) $$\rho \frac{\partial S_{2}^{k}}{\partial t}=k_{a2}\theta c-k_{d2}\rho S_{2}^{k}-\phi_{k2}$$

where $\partial S_{1}^{k}$ and $\partial S_{2}^{k}$ are the sorbed concentration of the first and second kinetic sorption sites, respectively (M M^−1^); *k*_a1_ and *k*_a2_ are the attachment coefficients of the first and second fraction of kinetic sorption sites, respectively (T^−1^); *k*_d1_ and *k*_d2_ are the detachment coefficients of the first and second fraction of kinetic sorption sites, respectively (T^−1^); and $\phi_{k1}$ and $\phi_{k2}$ are the sink term for the first and second kinetic sorption that represent various reactions at the kinetic sorption sites (n L^−3^ T^−1^) respectively.

In the same way, it can be reduced to one kinetic site sorption model when the kinetic sorption rates on both site fractions are the same, or can be reduced to a chemical equilibrium model when both rates are equally high to be considered instantaneous. These models have been successfully used to describe VA transport with the presence of DOM [92,95,101,102].

#### 6.1.2. Colloid-Facilitated Contaminant Transport Models

Contaminant transport in soil occurs not only in dissolved form but also in association with moving colloids. This mechanism of contaminant migration has been reported for numerous contaminants in the literature, including heavy metals [22,140,141], pesticides [142,143], and pharmaceuticals [33,144,145]. Colloid-associated contaminants may migrate at a rate faster than the non-sorbing tracer [146], which contributes an essential pathway for rapid contaminant transport, especially for highly sorbing organic contaminants in natural soil and groundwater [22,34,147,148,149]. Therefore, any fate and transport model for strongly sorbing contaminants that does not account for colloid-facilitated transport may under predict their migration potentials [22,147].

As DOM colloids satisfy the prerequisite to facilitate the transport of VAs in subsurface soil [22,23,150], a few studies have modelled VA transport in association with DOM [33,34]. The conceptual models for the colloid-facilitated transport of contaminants in subsurface soil and their mathematical descriptions have been presented in previous studies [22,23,150,151]. These models were built on the existing classical advection−dispersion equation (ADE) for water and solute transport based on the Richards equation. Flury and Qiu [147] provided a comprehensive review of the colloids and colloid facilitated transport of contaminants in the vadose zone. Here, we give a brief description of the model components and a summary of recent applications for VA transport prediction.

The one-dimensional form of the mass balance equations for the total contaminant transport in association with colloids under variably-saturated conditions is given as [22,150]:

(12) $$\frac{\partial \theta C}{\partial t}+\rho \frac{\partial S}{\partial t}+\frac{\partial A_{\mathrm{aw}}\Gamma }{\partial t}+\frac{\partial \theta_{w}C_{c}S_{\mathrm{mc}}}{\partial t}+\rho \frac{\partial S_{c}S_{\mathrm{ic}}}{\partial t}+\frac{\partial A_{\mathrm{aw}}\Gamma_{c}S_{\mathrm{mc}}}{\partial t}\;\;=\frac{\partial }{\partial z}\left(\theta D\frac{\partial C}{\partial z}\right)-\frac{\partial qC}{\partial z}+\frac{\partial }{\partial z}\left(\theta_{c}D_{c}\frac{\partial C_{c}S_{\mathrm{mc}}}{\partial z}\right)-\frac{\partial q_{c}C_{c}S_{\mathrm{mc}}}{\partial z}+R$$

where *C*, *S*, and *T* are the concentration of contaminant in the liquid phase (M L^−3^), sorbed concentration on solid-phase (M M^−1^), and air−water interphase (M M^−2^), respectively; *S*_mc_, *S*_ic_, and *S*_ac_ are concentrations of contaminant sorbed to mobile colloids in liquid-phase, immobile colloids on solid phase, and immobile colloids on the air−water interphase, respectively (M n^−1^); *D* and *q* are the dispersion coefficient (L^2^ T^−1^) and volumetric water flux (L T^−1^), respectively; and *R* represents contaminant reactions and degradation in all phases (M L^−3^ T^−1^).

### 6.2. Model Applications 

The presence of DOM can lead to changes in the number, nature, and reactivity of the sorption sites in the soil matrix domain, and thus complicate the transport of VAs in the soil. The chemical nonequilibrium models can be used to characterize both the transport behaviour and the sorption−desorption mechanism of antibiotics in homogeneous transport media through various conceptualisations, as described above (Equations (1)–(11); Table 3). For instance, in a column study, the competition of HA for available sorption sites on sand was evidenced in the transport of tetracycline, and the breakthrough curve (BTCs) of tetracycline can be satisfactorily described by the two sorption site model [101]. With increasing the HA concentration from 0 to 80 mg L^−1^, the fraction of equilibrium sorption site (*f*_e_) decreased from 0.185 to 0.133, while the kinetic rate constant (*α*_k_) increased from 1.91 to 3.72 d^−1^, which suggest a significant expansion of the nonequilibrium sorption sites (1 − *f*_e_) in the presence of DOM. Similarly, competitive sorption of DOM with various other antibiotics such as chlortetracycline, tylosin, sulfamethazine, and nalidixic acid was found to be responsible for the enhanced mobility of the antibiotics observed [92,95]. The fitting results of the antibiotic BTCs with the two sorption site model showed that, upon DOM addition, the mobility of chlortetracycline was greatly enhanced in both the surface and subsurface soils, while increased mobility of tylosin and sulfamethazine was observed only in surface soil columns, which had higher organic matter contents relative to the corresponding subsurface soil columns [95]. For nalidixic acid transport upon the addition of DOM, it was found that parameter *α*_k_ increased from 0.00095 to 0.05800 h^−1^, and the fitted *K*_d_ reduced from 39.77 to 7.60 cm^3^ g^−1^, which revealed facilitated transport and nonlinear kinetic binding of nalidixic acid on the mineral surface [102]. In another study on tetracycline transport, one kinetic sorption site model was used, and decreases in the attachment rate (*k*_att_) (from 1.135 to 0.665 min^−1^) and saturated sorption capacity (*S*_max_) (from 2.990 to 2.617 mg g^−1^) but increases in the detachment rate (*k*_det_) (from 0.001 to 0.010 min^−1^) were observed with the increasing HA concentration [92]. The colloid facilitated transport model could also be used to simulate the BTCs of SAs and manure DOM, as indicated by the normalised root mean square error (NRMSE) ranging from 0.01 to 0.39 [33]. It was found that manure DOM facilitated transport of florfenicol in homogeneously packed soil columns could be successfully described by both the two sorption site model and the colloid-facilitated contaminant transport model, with the latter performing slightly better [34] (Table 3). 

DOM derived from diverse sources may have different chemical properties, which complicate their interactions with Vas, as well as soil components. Accurate model simulation of VA transport in natural soils under the influences of DOM is essential for the assessment and control of VA pollution risk. So far, experimental and modelling studies on the effects of DOM−VA interactions on VA transport in soil have been mostly limited to a laboratory soil column scale. There is clearly a need for improved transport models applicable to heterogeneous complex field conditions and varying scenarios in farmland.

## 7. Conclusions and Future Research

Veterinary antibiotic contamination in farmland has attracted global attention due to the potential risks to the agroecosystem and the consequential impacts on human health. Even though some studies have investigated the transport of VAs, the unavoidable DOM−VA interactions made the investigation of VA transport in the presence of DOM a complex one. DOM can alter the sorption−desorption and leaching behaviour of VAs in farmland soil due to its high reactivity and mobility nature. The consequences of DOM−VA interactions on the environmental fate and risk of VAs have two facets. First, DOM−VA binding interactions can increase the persistence of VAs in soil through complexation and co-sorption on the soil matrix. This could increase the potential risk of antibiotic contamination to human health and may induce the development of ARGs in the environment. Second, DOM−VA interactions can enhance the leaching and transport of some VAs via competitive sorption that promotes VA desorption from soils or through the facilitated transport of VAs attached to mobile DOM. Adequate knowledge about the fate and transport of VAs under the influence of DOM derived from various soil amendments and organic fertilisers of diverse origins is crucial for the VA pollution risk assessment. Moreover, some measures, such as pre-treating manure to reduce antibiotic content by composting or anaerobic digestion, adding to manure or soil some sorbents or specific microorganisms, which can degrade VAs, could be considered as potential solutions to the antibiotic pollution problem in manured farmland.

Spectroscopic investigation of DOM−VA interactions can provide molecular evidence for understanding the mobility of VAs in soil. Nevertheless, effects of DOM−VA interactions on the transport of VAs under field conditions are still poorly understood. Therefore, we recommend the incorporation of DOM−VA interaction studies into simulation of VA transport. In addition, the significant variability in chemical properties of DOM derived from diverse sources and their potential to alter the fate of VAs in soil need to be investigated in the future. It is essential to consider undisturbed soil columns and lysimeters instead of repacked ones and to develop better transport models applicable to varying DOM−VA interaction scenarios in the field, where macropore preferential flow may prevail.

## Figures and Tables

**Table 1:** Effects of various DOM on sorption−desorption of VAs in soils.

DOM Type/Source	Extractant	Sorbate	Sorbent	Effect of DOM on Sorption/Desorption	Reference
Pig manure DOM	Water	Sulfadiazine	Various arable soils	Manure DOM decreased the sorption of sulfadiazine to soil due to competitive sorption.	[75]
Commercial humic acid (HA)	NaOH solution	Sulfamethoxazole	Crop straw biochar	HA intensified the sorption and desorption of sulfamethoxazole, and the dominant mechanism depended on HA concentration, sorbate properties, and the adlayer effects.	[76]
Soil organic matter	NA	Sulfanilamide	Soil	Sulfanilamide binding increased with increasing the soil organic matter polarity.	[77]
Manure and compost DOM	NA	Sulfamethoxazole	Soil	Sulfamethoxazole extractability decreased with increasing the complexity of co-extracted DOM over a wide incubation time.	[78]
Peak soil	NaOH solution	Sulfamethazine	Humin	Humin increased sulfamethazine sorption, and the sorption strength increased with the progressive removal of ash, O-alkyl carbon, lipid, and lignin components from humin.	[79]
Commercial DOM	CaCl_2_ solution	Sulfamethazine	Crop straw biochar	Sulfamethazine sorption to biochar decreased with increasing the HA concentration through surface modification, and competitive sorption/pore blockage, but the effects depended on the chemical properties of DOM, biochar properties, and nature of antibiotic species.	[48]
Soil HA	NA	Tetracycline and Clarithromycin	DOM	Tetracycline and clarithromycin strongly bound to DOM, but the solution pH and ionic strength influenced the binding interaction.	[80]
Soil DOM	CaCl_2_ solution	Tetracycline	Arable field topsoil	Presence of DOM caused significant sorption by enhancing the transfer of tetracycline from liquid phase to solid soil particles.	[81]
Plant and chicken manure derived DOM	Water	Oxytetracycline	Sediment	Plant-origin DOM (mainly humus-like) promoted oxytetracycline sorption, while chicken DOM (mainly protein-like) inhibited the sorption of oxytetracycline.	[82]
Wastewater treatment plant effluent DOM	NA	Sulfonamides (sulfapyridine, sulfameter, and sulfadimethoxine) and trimethoprim	Agricultural soils	Presence of DOM lowered the linear distribution coefficient (*K*_d_) of the tested antibiotics in soils.	[46]
Exogenous DOM	CaCl_2_ solution	Sulfamethoxazole, sulfamethazine, and sulfadiazine	Soil with/without biochar amendment	DOM decreased the sorption of the three target antibiotics to biochar amended soils.	[83]
Commercial DOM	NA	Tylosin and sulfamethazine	HA	Tylosin and sulfamethazine were strongly sorbed to HA through cation exchange and π-π EDA interaction mechanisms.	[84]
Fresh and aged soil-biochar mixture	CaCl_2_ solution	Oxytetracycline and florfenicol	Soil with/without biochar amendment	Biochar released DOM reduced oxytetracycline sorption to soil through competitive sorption while it increased florfenicol sorption through hydrophilic partitioning.	[85]
Wastewater DOM	NA	Sulfadimidine	DOM solutions with/without ozonation	Strong complexation of sulfadimidine with DOM was enhanced by protein-like DOM components (tryptophan-like and tyrosine-like).	[86]
Surface water DOM	NA	Sulfamethoxazole and clarithromycin	Natural DOM	An insignificant binding interaction was observed for the antibiotics due to their lower hydrophobicity and weak acid groups.	[87]
Sediment derived HA	NaOH solution	Ofloxacin	DOM	Nonlinear binding interactions between ofloxacin and DOM dominated, which involved H-bonding, electrostatic interactions, and cation exchange.	[88]
Phytoplankton and macrophyte derived DOM	Water	Sulfamethazine	Goethite	An increasing concentration of DOM facilitated the sulfamethazine sorption on goethite, which was more significant in DOM dominated by protein-like substances.	[89]
Crop straw biochar derived DOM	Water	Sulfamethoxazole and chloramphenicol	Biochars	DOM concentration modulated antibiotic sorption depending on the proportions of fulvic and HA-like DOM components.	[90]
Composted biosolid derived DOM	Water	Sulfapyridine	Agricultural soils	Co-introduction of DOM with sulfapyridine significantly reduced its sorption to soils, and DOM precoated soil exhibited both cumulative sorption and reduced sorption.	[25]
Poultry litter DOM	Water	Sulfamethazine	Soils of different land use types	Competitive interactions occurred between sulfamethazine and DOM (>1000 Da), leading to reduced sulfamethazine sorption to soil.	[91]
Wastewater effluent DOM	NA	Tetracycline	Soil	Tetracycline sorption to soil decreased with increasing the HA concentration due to the high mobility and competitive sorption.	[92]
Decayed plant and composted manure derived DOM	Water	Oxytetracycline	Sediments	Decayed plant-derived DOM promoted oxytetracycline sorption, while manure DOM exhibited inhibitory effects. DOM concentration modulated antibiotic sorption.	[93]
Commercial HA	NaOH solution	Ofloxacin/flumequine	Kaolinite	Presence of DOM enhanced the co-precipitation of ofloxacin/flumequine from aqueous phase, but the effects varied depending on pH.	[94]

Note: NA = not applicable.

**Table 2:** A summary of the findings on DOM−VA interactions using various spectroscopic techniques.

Interaction	DOM Concentration	VA Concentration	Spectroscopic Method	Finding	Reference
Fluoroquinolones (ciprofloxacin, enoxacin, ofloxacin, norfloxacin) and DOM	0–2.8 mg L^−1^	1.8 mg L^−1^	Nuclear magnetic resonance (NMR) spectroscopy, fluorescence quenching, UV−VIS spectroscopy, and Fourier transform infrared (FTIR) spectroscopy	A static and exothermic binding interaction occurred between FQs and humic acid (HA). H-bonding, electrostatic effect, van der Waals force, and π−π stacking were involved. The aromatic ring and double bond proton were the central binding region for HA. The main DOM functional groups involved were O-H, C-H, -COOH, and N-H.	[31]
Sulfamethazine and DOM	20 mg-C L^−1^	0–25 mg L^−1^	Fluorescence excitation−emission matric spectroscopy combined with parallel factor analysis (EEM-PARAFAC)	Static fluorescence quenching dominated the sulfamethazine−DOM interaction. The protein-like component of DOM formed the strongest binding interaction with sulfamethazine with about 95–100% quenched, while the humic-like quenched was about 68–86%. The interaction of sulfamethazine with DOM components followed the order: tryptophan- > tyrosine- > humic-like component. The binding affinity of sulfamethazine to protein-like and humic-like DOM components were 2.75–4.25 and 2.06–2.78, respectively.	[24]
Fluoroquinolones (ciprofloxacin, enoxacin, fleroxacin, levofloxacin, norfloxacin, and ofloxacin) and DOM (HA)	0–2.5 mg L^−1^	3.0 × 10^−5^ M	Fluorescence quenching	Static quenching occurred in DOM−FQ interactions, and equilibrium binding constants were <1 for all the FQs, indicating a weak binding interaction due to the high solubility nature of the FQs and the weak H-bonding.	[24]
Tetracycline and DOM	15 mg L^−1^	0–60 µmol L^−1^	Fluorescence EEM-PARAFAC, synchronous fluorescence spectra combined with two-dimensional correlation spectroscopy (2D-COS), UV−VIS spectroscopy, and FTIR spectroscopy	Static quenching followed by complexation occurred in DOM−tetracycline interactions in the order of tryptophan-like > tyrosine-like > humic-like component. Amide I and II, aromatics, and aliphatics were the main functional groups responsible for the interactions.	[89]
Ofloxacin and dissolved HA	25 mg-C L^−1^	50–200 mg L^−1^	Fluorescence quenching and FTIR spectroscopy	The interaction between ofloxacin and dissolved HA involved a combination of static and dynamic quenching. The dominant mechanism depended on the abundance of carboxyl groups in dissolved HA. Electrostatic interactions and cation exchange were the main mechanisms.	[29]
Four antibiotics (roxarsone, sulfaquinoxaline, oxytetracycline, and erythromycin) and DOM	−	0.5–4.0 mg L^−1^	Fluorescence EEM-PARAFAC	DOM−antibiotic interaction caused significant quenching of DOM fluorophore and eventual complexation reaction. The interaction followed the order: tyrosine ≥ tryptophan > HA component.	[117]
Tetracycline and DOM	−	5–50 mg L^−1^	Fluorescence EEM, synchronous fluorescence, 2D-COS, and FTIR spectroscopy	DOM−tetracycline interaction led to static fluorescence quenching. The binding order was: tryptophan-like > tryptophan-like > humic-like substance. Non-fluorescence components, including polysaccharide-like substance and aliphatic compound, were also involved.	[81]
Tetracyclines (Tetracycline, oxytetracycline and chlortetracycline) and DOM	534.4 mg L^−1^	0.17–0.26 mg L^−1^	Fluorescence EEM combined with the fluorescence regional integration (FRI) method, FTIR spectroscopy, and UV−VIS spectroscopy	Larger molecular fractions of DOM significantly influenced the phase distribution of TCs due to the variation in their physiochemical properties. Surface complexation, hydrogen bonding, and electrostatic interactions were dominant mechanisms. The complexation with TCs followed the order: fulvic > protein-like> HA-like component.	[27]
Sulfamethazine and HA	0.13–2.88 mg L^−1^	0.5 mg L^−1^	Surface plasmon resonance combined with isothermal titration microcalorimetry technologies	Stable and strong binding interaction between HA and sulfamethazine occurred through hydrogen bonding, electrostatic interaction, and hydrophobic interactions.	[32]
Oxytetracycline, sulfadiazine, and HA	200 mg L^−1^	1.5–9.0 mg L^−1^	Fluorescence EEM	Interactions between HA and antibiotics led to the formation of complexes through H-bonding and van der Waals force, as reflected by the observed static quenching.	[82]
Ofloxacin and DOM	5–40 mg L^−1^	3 mg L^−1^	Fluorescence quenching technology, elemental characterization, and infrared spectrum	The DOM−ofloxacin interaction involved static and dynamic quenching. The binding interaction increased with decreasing the HA polarity. DOM hydrophobicity played a significant role in the interaction.	[26,88]
Tetracycline and HA	5–50 mg L^−1^	0–20 mg L^−1^	Fluorescence EEM combined with FRI method and molecular docking	HA formed strong complexes with tetracycline through electrostatic forces and proton-affinity sites. Other intermolecular interactions involved were hydrogen bond, van der Waals, electrostatics, and torsional forces.	[92]
Tetracycline and HA	0.25 g L^−1^	5 µM	FTIR, NMR, and 2D-COS	Tetracycline strongly bound to HA. Carboxyl and phenolic hydroxyl groups in HA and -N(CH_3_)_2_ groups of tetracycline were engaged in the interaction. The binding interaction reduced the degradation of tetracycline.	[125]
Enrofloxacin, DOM (humic acid and fluvic acid) and montmorillonite	1 g L^−1^	27.8 µM	Attenuated total reflection-Fourier transform infrared spectroscopy and 2D-COS	Enrofloxacin was sorbed to montmorillonite through cation exchange, proton transfer, electrostatic interaction, H-bonding, and π−π interactions, depending on solution pH.	[126]
Tetracycline and HA	2.3, 23, and 46 mg-C L^−1^	5–100 mg L^−1^	X-ray diffraction and FTIR spectroscopy	HA−tetracycline interaction reduced tetracycline mobility through complexation. The main mechanism involved was electrostatic interaction between tetracycline (cationic or zwitterionic species) and carboxylic groups in HA.	[30]
Sulfamethoxazole, clarithromycin, and DOM	0–15 mg L^−1^	10 and 20 μg mL^−1^	Cellulose ester dialysis membranes separation and LC-MS/MS analysis	DOM-binding through hydrophobic interaction was not observed for the two antibiotics.	[87]

**Table 3:** Applications of models in simulating the transport of VAs as affected by DOM.

Transport Species	Injection Concentrations and Transport Conditions				Transport Model and Parameters						Reference
	DOM	VA	Media	DOM Injection Method	Colloid Facilitated Transport Model						
					*k*_dec_ (s^−1^)	$k_{a}^{m}$ (L mol^−1^ s^−1^)	$k_{d}^{m}$ (s^−1^)	$k_{a}^{im}$ (L mol^−1^ s^−1^)	$k_{d}^{im}$ (s^−1^)	NRMSE	
Pig manure DOM and sulfadiazine	8–115 mg L^−1^	250 μg L^−1^	Loamy sand soil	Co-transport	1.98 × 10^−9^–1.78 × 10^−5^	3.81 × 10^−1^– 8.58 × 10^−3^	7.62 × 10^−5^– 7.22 × 10^−4^	3.30 × 10^−7^– 7.14 × 10^−1^	1.18 × 10^−6^– 6.55 × 10^−5^	0.02–0.05	[33]
Pig manure DOM and sulfamethoxypyridazine						4.14 × 10^−4^– 1.33 × 10^−2^	1.49 × 10^−5^– 5.14 × 10^−4^	1.38 × 10^−3^– 5.23 × 10^−3^	1.56 × 10^−7^– 6.62 × 10^−6^	0.01–0.09	
Pig manure DOM and sulfamoxole						1.93 × 10^−4^– 2.82 × 10^−1^	2.01 × 10^−7^– 7.18 × 10^−4^	3.00 × 10^−6^– 7.14 × 10^−1^	1.99 × 10^−3^– 1.56 × 10^−6^	0.02–0.23	
					Advection dispersion equation coupled with two-site nonequilibrium sorption model						
					*K*_d_ (*K*_f_ *)	*β* (–)	*f*_e_ (–)	*α*_k_	*D*	R^2^	
Leonardite humic acid (HA) and nalidixic acid	5–50 mg L^−1^	10 µM	Goethite-coated sand	Pre-sorbed and co-transport	3.72–34.55 * cm^3^ g^−1^	0.74–1.15	0.6	0.00766–0.01859 h^−1^	–	0.989–0.999	[102]
Diluted dairy manure DOM and florfenicol	85 mg L^−1^	100 μg mL^−1^	Silt loam	Co-transport	0.84 * cm^3^ g^−1^ 0.45 cm^3^ g^−1^	0.75 –	0.61–0.51	0.13–0.19 h^−1^	0.31 cm^2^ h^−1^	0.984–0.992	[34]
Dairy manure DOM and chlortetracycline	21–63 mg L^−1^	0.07–0.58 × 10^−3^ M	Sandy loam soil	Co-transport	158–159 L kg^−1^	–	0.018–0.053	0.027–0.085 h^−1^	9.14–26.30 cm^2^ h^−1^	0.91–0.92	[95]
Dairy manure DOM and tylosin					4.04–8.19 L kg^−1^	–	0.14–0.32	0.10–0.29 h^−1^	7.76–69.70 cm^2^ h^−1^	0.89–0.91	
Dairy manure DOM and sulfamethazine					0.30–0.45 L kg^−1^	–	0.21–0.56	0.071–0.68 h^−1^	5.19–30.40 cm^2^ h^−1^	0.99	
Soil HA and tetracycline	20–80 mg L^−1^	10 mg L^−1^	Fine-to-medium-grain sand	Co-transport	7.29–11.80 L kg^−1^	0.156–0.235	0.133–0.214	2.22–3.89 d^−1^	0.483 cm^2^ d^−1^	0.998	[101]
Soil HA and pyrene					3.13–6.43 L kg^−1^	0.462–0.566	0.432–0.553	3.29–5.09 d^−1^	0.515 cm^2^ d^−1^	0.998	
					Advection dispersion equation coupled with two kinetic site model						
					*K*_d_ (L kg^−1^)	*k*_a1_ (×10^−2^ h^−1^)	*k*_d1_ (×10^−2^ h^−1^)	*k_a_*_2_ (×10^−2^ h^−1^)	–	–	
HA and tetracycline	–	2 mg L^−1^	Quartz sand	Pre-sorption to soil and co-transport	25.40	10.30–52.60	0.90–17.60	24.10–86.6	–	–	[98]
HA and ciprofloxacin					34.40	6.40–27.00	1.33–16.90	15.30–81.70	–	–	
					Advection dispersion model coupled with one kinetic site sorption model						
					*K*_d_	*k*_att_ (min^−1^)	*k*_det_ (min^−1^)	*S*_max_ (mg g^−1^)	*D* (cm^2^ min^−1^)	R^2^	
Soil HA and tetracycline	5–20 mg L^−1^	20 mg L^−1^	Fine to medium grained soils	Co-transport	–	0.295–1.135	0.001–0.010	0.294–2.990	0.415–0.461	0.913–0.974	[92]

Notes: *k*_dec_ is the colloid decay rate constant; $k_{a}^{m}$ and $k_{d}^{m}$ are the attachment and detachment rate coefficients for the mobile colloids, respectively; $k_{a}^{im}$ and $k_{d}^{im}$ are the attachment and detachment rate coefficients for the immobile colloids, respectively. *K*_d_ is the linear sorption coefficient; *K*_f_ is the Freundlich isotherm sorption coefficient; *β* is an empirical parameter that characterises the degree of nonlinearity; *f_e_* is the fraction of instantaneous equilibrium sorption (Type-1) sites; *α*_k_ is the first-order rate coefficient associated with the kinetic site; *D* is the dispersion coefficient. *k*_a1_ and *k*_a2_ are the first order attachment rate of Type 1 site and Type 2 site, respectively; *k*_d1_ is the first-order detachment rate of Type 1 site. *k*_att_ and *k*_det_ are the first-order attachment rate and detachment rate, respectively; *S*_max_ is the saturated sorption capacity. The number followed by * represents the value of *K*_f_.

## Data Availability

Not applicable.

## Supplementary Materials

The following are available online at https://www.mdpi.com/article/10.3390/ijerph19031702/s1, Table S1. Nature and parameters of DOM from various sources.
