# Supplementary material for: Transport of Veterinary Antibiotics in Farmland Soil: Effects of Dissolved Organic Matter
Source: Int J Environ Res Public Health. 2022 Feb 2;19(3):1702. doi: 10.3390/ijerph19031702 (PMC8834935; doi:10.3390/ijerph19031702)
Supplement: Supplementary file 1 [file ijerph-19-01702-s001.zip › ijerph-1560210-supplementary.pdf]

Table S1. Nature and parameters of DOM from various sources.

DOM Source	Sample type	DOC content	EEM-PARAFAC			Surrogate parameters of DOM/Fluorescence indices					Reference
			Ex/Em (nm)	Components	Proportion/order	$SUVA_{254}$	Slope R	FI	HIX	BIX	
Grassland soil amended with compost produced from urban green-waste	Soil solution	13.58–83.34 mg L^{-1}	330–350/ 420–440<br>240–260/ 420–480<br>270–280/ 300–340	C1 - humic-like substances<br>C2 - fulvic acid-like<br>C3 - protein like	C1 > C2 > C3	N/A	N/A	1.3	17.0–15.4	0.7	[1]
Agricultural soil and gravel aquifer systems	Extract with 2M KCl	6.90–14.2 mg L^{-1}	330–340 /410–460<br>270–290/ 320–360<br>270–280/ 294–302	C1- fulvic-like<br>C2 - tryptophan like<br>C3 - tyrosine-like	C1 > C2 > C3	NA	0.46–0.61	1.42	9.06	0.54	[2]
Crop straws	Extract with water	779–857 mg L^{-1}	NA	NA	NA	0.728	N/A	1.5–1.7	0.23–0.27		[3]
Agricultural watersheds	Runoff	2.50–9.8 mg L^{-1}		C1 - humic-like<br>C2 - protein-like	C1 > C2	N/A	0.01–0.02	N/A	N/A		[4]
Paddy soils amended with biochar produced from wheat straw	Extract with water	0.06–1.45 g kg^{-1}	245 (265)/380 nm<br>220/410(420)<br>260(280) /440(480)	C1 - tryptophan-like<br>C2 - UVA humic acid-like<br>C3 - UVC humic acid-like	11–38%<br>42–48%<br>19–48%	0.11–4.58	3.69–5.95	1.82–2.46	0.82–2.46	0.29–0.84	[5]
Various cropped and natural Chinese soils	Extract with water	0–0.36 mg g^{-1}	265/460<br>230/400<br>320(250) /400<br>230(275) /330<br>220/330	C1 - fulvic-like<br>C2 - fulvic-like<br>C3 - humic-like<br>C4 - tryptophan-like<br>C5 - peptide-like	16–48%<br>15–41%<br>9–24%<br>7–24%<br>0–25%	1.10–2.05	N/A	1.57–1.91	0.70–0.92	0.61–0.88	[6]
Farmland soils amended with	Extract with water	83.99–144.2	425/522	C1 - fulvic acid-like sub-	C2 > C1 >C3	N/A	N/A	0.84–0.96	0.99–1.03	5.11–6.10	[7]

wheat straw biochar		7 mg kg^{-1}		stances							
			350/426	C2 - humic acid-like substances							
			205/324	C3 - tryptophan-like substances							
Loamy cropland soil	Runoff and fracture flow	0.40–84 mg L^{-1}	270(380)/480	C1 - terrestrial/autochthonous UVA humic-like	N/A	2.9–10	N/A	1.52–1.61	0.87–0.91	N/A	[8]
			250(320)/410	C2 - anthropogenic UVA humic-like							
Semiarid agricultural soil	Extract with Water	12.85–27.90 mg L^{-1}	<240–250/390–440	C1 - terrestrial humic-like	C4 > C2 > C3 > C1	0.76–1.96	N/A	N/A	2.22–2.98	N/A	[9]
			<240–275/455–540	C2 - terrestrial humic-like							
			<240/303–312	C3 - monolignol-like							
			<240/303–312	C4- protein-/Tannin-like							
Agricultural soils amended with wheat straw	Extract with water	0.13–0.34 g kg^{-1}	340/435	C1 - UVA/UVC humic-like	42–67%	1.04–7.30	1.0–3.30	1.12–1.3	1.04–1.14	N/A	[10]
			280(420)/520	C2 - fulvic acid-like	26–32%						
			215(270)/340	C3 - tryptophan-like	6–27%						
Wheat straw-derived biochar	Extract with water, alkaline and acid	435–5000 mg kg^{-1}	340/426	C1 - terrestrial humic-like	10–61%	0.2–78.9	1.8–4.4	N/A	0.99–1.92	N/A	[11]
			395/474	C2 - high molecular weight humic-like	20–66.4%						
			465/518	C3 - fulvic acid-like	9–37.1%						
			380(518)/590	C4 - humic-like	7–17.8%						
			300(375)/490	C5 - UVA humic acid-like	~10.80%						

			200/294	C6 - tryptophan-like	5–12.5%						
Soil and sediments of agricultural land	Extract with water	6.90–30.23 mg L^{-1}	270/460<br>250/400<br>220/426	C1 - traditional humic-like<br>C2 - traditional humic-like<br>C3 - humic-like	N/A<br>N/A<br>N/A	3.07–4.70	N/A	1.50–1.70	7.00–11.10	0.50–0.65	[12]
Soils of riparian buffer wetland	Extract with water	0.07–0.27 g kg^{-1}	250–260/ 420–460<br>270–290/ 320–350<br>320–360/ 400–450<br>225–230/ 320–350	Peak A -humic-like<br>Peak B -Protein-like<br>Peak C -Humic-like<br>Peak T -Protein-like	N/A	N/A	N/A	1.79–2.54	>4	0.60–0.70	[13]
Paddy field soils under long-term chemical fertiliser, crop straw, manure, and manure compost amendments	Extract with water	330–760 mg kg^{-1}	250(300) /400–450<br>250(400(/450–500<br>300–350/ 350–400<br>250–300/ 300–350<br>250–300/ 350	C1 - humic-like<br>C2 - humic-like<br>C3 - microbial humic-like<br>C4 - tyrosine-like<br>C5 - tryptophan-like	38–53%<br>14–20 %<br>6–13%<br>14–29%<br>4–8%	0.5–2.0	N/A	N/A	N/A	N/A	[14]
Straw of canola and oilseed rape	Extract with water	779–857 mg L^{-1}	N/A	N/A	N/A	0.97–3.13	N/A	1.5–1.8	0.80–1.20		[15]
Cattle manure and corn straw compost	Extract with water	N/A	215–225 (275–280)/330(330)<br>225(280) /340<br>240(320) /420	C1 - tryptophan-like<br>C2 - humic-like<br>C3 - terrestrial humic-like	N/A	0.15–0.55	0.20–0.37	N/A	N/A	N/A	[16]
Frequently submerged agricultural soils	Extract with water	7.80–16%	N/A	N/A	N/A	1.07–1.50	N/A	1.54–1.72	3.66–7.91	0.62–0.92	[17]
Fresh biochar and long-term biochar amended agricultural	Extract with water	14.14–84.18 mg L^{-1}	N/A	N/A	N/A	1.23–4.68	1.28–5.70	1.13–1.72	5.33–14.65	N/A	[18]

soils											
Biochar derived from chicken, pig, cow, and sheep manure	Extract with water	4.57–18.70 g kg^{-1}	310/404	C1 - marine humic-like	8–41%	4.75–7.18	N/A	N/A	7.0–25.87	N/A	[19]
			350/436	C2 - UVA humic-like	8–33%						
			340/383	C3 - microbial by-product	10–43%						
			260 (400)/490	C4 - UVC + UVA humic-like	7–15%						
Composted pig and cattle manure with sawdust and corn stalks	Extract with water	20–85 g kg^{-1}	230(275)/330	C4 - tryptophan-like	7–24	N/A	N/A	N/A	N/A	N/A	[20]
			220/330	C5 - peptide like	0–25%						
			245/399nm	C3 - soluble microbial by-product-like	26.30–40%						
			245/400nm	C4 - fulvic-like	10.9–18.10%						
			305/412 nm	C5 - humic-like materials	12.5–15.30%						
Biochars produced from soybean, stover, garlic stem, rice husk, tea waste, perilla, wood pine chip, and oak wood	Extract with water	0.40–659 mg L^{-1}	260(340)/430	C1 - humic-like	6–37%	N/A	N/A	N/A	N/A	N/A	[21]
			220(280)/370	C2 - protein-/tannin-like	5–100%						
			260/440	C3 - Fulvic acid-like	17–67%						
			270(350) 490	C4 - terrestrial humic-like	9–37%						
Biochar produced from sawmill waste feedstocks	Extract with water	14.99–37.20 mg L^{-1}	<250 (280)/390	C1 - microbial humic-like	27–38%	0.70–6.0	N/A	N/A	N/A	N/A	[22]
			250(320)/435	C2 - fulvic/humic-like	22–31%						
			260/315	C3 - protein-like (tyrosine-like)	25–45%						
			290 (350)/525	C4 - soil humic-like	4.5–7.0%						

Biochars produced from sewage, soybean, rice, and peanut	Extract with water	17.60–53.8 mg L^{-1}	230–250/ 390–425	Peak A - humic acid/marine humic	N/A	0.1~1.25	N/A	N/A	N/A	N/A	[23]
			305–310/ 405–425	Peak B - humic acid/marine humic substances							
Biochars produced from almond shell, broiler litter, lignin, cottonseed hull, and pecan shell	Extract with water	0–15692 mg L^{-1}	240(300) /420	C1 - fulvic-like	8–83%	N/A	N/A	N/A	N/A	N/A	[24]
			250 (300)/430	C2 - UVC humic-/ marine humic-like	3–35%						
			250 (350)/470	C3 - UVC and UVA humic-like	4–43%						
			340/380	C4 - microbial decomposition products	3–59%						
			220(280) /340	C5 - protein-like moieties	3–60%						
Agricultural soils amended with biochar	Extract with $CaCl_2$, $Na_4P_2O_7$, and toluene/methanol	156–201 mg L^{-1}	240(320–340)/400–420	C1 - protein-like	15–53%	N/A	N/A	N/A	N/A	N/A	[25]
			260–280 (340)/460	C2 - humic-like	18–27%						
			220(280) /340	C1 - protein-like	17–63%						
			200/294	C6- tryptophan-like	5–12.5%						
Leaf litters from *Populus simonii*, *Artemisia desertorum*, and *Salix cheilophila*	Extract with water	1588–2544 mg L^{-1}	210–245/390–425	C1 - fulvic acid-like	N/A	N/A	N/A	N/A	N/A	N/A	[26]
			295–315 /415–435	C2 - humic acid-like	N/A						
			275–280 (220–225)/300–320 (295–310 nm)	C3 - protein-like	N/A						
			275 (225)/345 (345)	C4 - protein-like	N/A						

BIX: Biological index is an indicator of the relative contribution of the recently microbially produced DOM, i.e., the ratio of albuminoid and biological components; FI: Fluorescence index is used as an indicator of DOM source to infer the relative microbial (> 1.9) or terrestrial plant (< 1.4) contribution to DOM; HIX: Humification index reflects the degree of humification, vital complexity and condensation (H/C ratios) of terrigenous contribution (> 10); Slope R: slope ratio is the ratio in the shorter UV wavelength region (275–295 nm) relative to that in the longer UV wavelength region (350–400 nm), reflecting molecular weight and source of DOM; $SUVA_{254}$: specific UV-visible absorbance indicates the aromaticity of DOM ($SUVA_{254}$ < 3 indicates the hydrophilic fractions, whereas $SUVA_{254}$ > 4 indicates the aromatic and hydrophobic fractions).

References

1. Musadji, N.Y.; Lemée, L.; Caner, L.; Porel, G.; Poinot, P.; Geffroy-Rodier, C. Spectral characteristics of soil dissolved organic matter: Long-term effects of exogenous organic matter on soil organic matter and spatial-temporal changes. *Chemosphere* **2020**, *240*, 124808.
2. Tye, A.M.; Lapworth, D.J. Characterising changes in fluorescence properties of dissolved organic matter and links to N cycling in agricultural floodplains. *Agr. Ecosyst. Environ.* **2016**, *221*, 245-257.
3. Jia, W.; Zhao, X.-H.; Zhao, Y.-Y.; Xu, J.-Y.; Ming, J.-J.; Cai, M.-M., Hu, C.-X. Variation in spectral characteristics of dissolved organic matter derived from rape straw of plants grown in Se-amended soil. *J. Integr. Agr.* **2020**, *19*, 1876-1884.
4. Eckard, R.S.; Pellerin, B.A.; Bergamaschi, B.A.; Bachand, P.A.M.; Bachand, S.M.; Spencer, R.G.M.; Hernes, P.J. Dissolved Organic Matter Compositional Change and Biolability During Two Storm Runoff Events in a Small Agricultural Watershed. *J. Geophys. Res-Biogeo.* **2017**, *122*, 2634-2650.
5. Gao, J.; Shi, Z.; Wu, H.; Lv J. Fluorescent characteristics of dissolved organic matter released from biochar and paddy soil incorporated with biochar. *RSC Adv.* **2020**, *10*, 5785-5793.
6. Li, S.; Li, M.; Wang, G.; Sun, X.; Xi, B.; Hu, Z. Compositional and chemical characteristics of dissolved organic matter in various types of cropped and natural Chinese soils. *Chem. Biol. Technol. Ag.* **2019**, *6*, 20.
7. Zhang, A.; Zhou, X.; Li, M.; Wu, H. Impacts of biochar addition on soil dissolved organic matter characteristics in a wheat-maize rotation system in Loess Plateau of China. *Chemosphere* **2017**, *186*, 986-993.
8. Xian, Q.; Li, P.; Liu, C.; Cui, J.; Guan, Z.; Tang, X. Concentration and spectroscopic characteristics of DOM in surface runoff and fracture flow in a cropland plot of a loamy soil. *Sci. Total Environ.* **2018**, *622-623*, 385-393.
9. Romero, C.M.; Engel, R.E.; D'Andrilli, J.; Chen, C.; Zabinski, C.; Miller, P.R.; Wallander, R. Bulk optical characterization of dissolved organic matter from semiarid wheat-based cropping systems. *Geoderma* **2017**, *306*, 40-49.
10. Gao, J.; Lv, J.; Wu, H.; Dai, Y.; Nasir, M. Impacts of wheat straw addition on dissolved organic matter characteristics in cadmium-contaminated soils: Insights from fluorescence spectroscopy and environmental implications. *Chemosphere* **2018**, *193*, 1027-1035.
11. Li, M.; Zhang, A.; Wu, H.; Liu, H.; Lv, J. Predicting potential release of dissolved organic matter from biochars derived from agricultural residues using fluorescence and ultraviolet absorbance. *J. Hazard. Mater.* **2017**, *334*, 86-92.
12. Liu, C.; Li, Z.; Berhe, A.A.; Xiao, H.; Liu, L.; Wang, D.; Peng, H.; Zeng, G. Characterizing dissolved organic matter in eroded sediments from a loess hilly catchment using fluorescence EEM-PARAFAC and UV–Visible absorption: Insights from source identification and carbon cycling. *Geoderma* **2019**, *334*, 37-48.
13. Wang, Y.-L.; Yang, C.-M.; Zou, L.-M.; Cui, H.-Z. Spatial distribution and fluorescence properties of soil dissolved organic carbon across a riparian buffer wetland in Chongming Island, China. *Pedosphere* **2015**, *25*, 220-229.
14. Wu, H.; Kida, M.; Domoto, A.; Hara, M.; Ashida, H.; Suzuki, T.; Fujitake, N. The effects of fertilization treatments and cropping systems on long-term dynamics and spectroscopic characteristics of dissolved organic matter in paddy soil. *Soil Sci. Plant Nutr.* **2019**, *65*, 557-565.
15. Wang, B.; Li, M.; Zhang, H.; Zhu, J.; Chen, S.; Ren, D. Effect of straw-derived dissolved organic matter on the adsorption of sulfamethoxazole to purple paddy soils. *Ecotox. Environ. Saf.* **2020**, *203*, 110990.
16. Chen, Y.; Jiang, Z.; Zhang, X.; Cao, B.; Yang, F.; Wang, Z.; Zhang, Y. Variation in the Humification Degree of Dissolved Organic Matter from Cattle Manure during Composting as Analyzed by Ultraviolet-Visible and Fluorescence Spectroscopy. *J. Environ. Qual.* **2017**, *46*, 1489-1499.

17. Jiang, T.; Kaal, J.; Liang, J.; Zhang, Y.; Wei, S.; Wang, D.; Green, N.W. Composition of dissolved organic matter (DOM) from periodically submerged soils in the Three Gorges Reservoir areas as determined by elemental and optical analysis, infrared spectroscopy, pyrolysis-GC–MS and thermally assisted hydrolysis and methylation. *Sci. Total Environ.* **2017**, *603-604*, 461-471.
18. Cai, W.; Du, Z.-L.; Zhang, A.-P.; He, C.; Shi, Q.; Tian, L.-Q.; Zhang, P.; Li, L.-P.; Wang, J.-J. Long-term biochar addition alters the characteristics but not the chlorine reactivity of soil-derived dissolved organic matter. *Water Res.* **2020**, *185*, 116260.
19. Peng, N.; Wang, K.; Tu, N.; Liu, Y.; Li, Z. Fluorescence regional integration combined with parallel factor analysis to quantify fluorescencent spectra for dissolved organic matter released from manure biochars. *RSC Adv.* **2020**, *10*, 31502-31510.
20. Zhang, F.; Li, Y.; Xiong, X.; Yang, M.; Li, W. Effect of Composting on Dissolved Organic Matter in Animal Manure and Its Binding with Cu. *Sci. World J.* **2012**, *2012*, 289896.
21. Rajapaksha, A.U.; Ok, Y.S.; El-Naggar, A.; Kim, H.; Song, F.; Kang, S.; Tsang, Y.F. Dissolved organic matter characterization of biochars produced from different feedstock materials. *J. Environ. Manage.* **2019**, *233*, 393-399.
22. Jamieson, T.; Sager, E.; Guéguen, C. Characterization of biochar-derived dissolved organic matter using UV–visible absorption and excitation–emission fluorescence spectroscopies. *Chemosphere* **2014**, *103*, 197-204.
23. Tang, J.; Li, X.; Luo, Y.; Li, G.; Khan, S. Spectroscopic characterization of dissolved organic matter derived from different biochars and their polycylic aromatic hydrocarbons (PAHs) binding affinity. *Chemosphere* **2016**, *152*, 399-406.
24. Uchimiya, M.; Ohno, T.; He, Z. Pyrolysis temperature-dependent release of dissolved organic carbon from plant, manure, and biorefinery wastes. *J. Anal. Appl. Pyrol.* **2013**, *104*, 84-94.
25. Uchimiya, M.; Liu, Z.; Sistani K. Field-scale fluorescence fingerprinting of biochar-borne dissolved organic carbon. *J. Environ. Manage.* **2016**, *169*, 184-190.
26. Zhao, L.; Du, C.; Zhang, Q.; Sun, C.; Wang, S.; Luo, S. The ultraviolet–visible absorbance and fluorescence characterization of dissolved organic matter derived from the leaf litter of Populus simonii, Artemisia desertorum, Salix cheilophila, and Populus tomentosa. *Environ. Sci. Pollut. Res.* **2020**, *27*, 36439-36449.
